# Modulatory Effects of *Satureja montana* L. Essential Oil on Biofilm Formation and Virulence Factors of *Pseudomonas aeruginosa*

**DOI:** 10.3390/ph18091269

**Published:** 2025-08-26

**Authors:** Gordana Maravić-Vlahoviček, Marija Kindl, Klara Andričević, Sonja Obranić, Sanda Vladimir-Knežević

**Affiliations:** 1 Department of Biochemistry and Molecular Biology, Faculty of Pharmacy and Biochemistry, University of Zagreb, A. Kovačića 1, 10000 Zagreb, Croatia; klara.andricevic1@gmail.com; 2 Department of Pharmacognosy, Faculty of Pharmacy and Biochemistry, University of Zagreb, Trg Marka Marulića 20, 10000 Zagreb, Croatia; marija.kindl@gmail.com; 3 Department of Nursing, University Centre Varaždin, University North, 104. Brigade 1, 42000 Varaždin, Croatia; sobranic@unin.hr

**Keywords:** *Satureja montana*, winter savory, essential oil, thymol, biofilm, swarming motility, proteolytic activity, pyocyanin

## Abstract

**Background**: Antimicrobial resistance is a major global health threat, particularly from pathogens such as *Pseudomonas aeruginosa*, known for forming biofilms and producing virulence factors that cause persistent infections. Essential oils (EOs) offer promising alternatives to conventional antimicrobial therapy due to their antimicrobial and antibiofilm properties. This study aimed to investigate the modulatory effects of a thymol-rich EO from *Satureja montana* L. on planktonic growth, biofilm formation, swarming motility, proteolytic activity and pyocyanin production of *P. aeruginosa* PAO1. **Methods**: The essential oil, isolated by hydrodistillation from *S. montana* aerial parts, was analysed by GC-MS. The minimum inhibitory concentration (MIC) of the EO and thymol was determined using the broth microdilution method. Sub-MICs were tested for planktonic growth and biofilm formation. Virulence was assessed by testing swarming motility, proteolytic activity and pyocyanin production. **Results**: The EO was characterised by a very high content of monoterpenes, with thymol dominating (56.47%). MIC for both EO and thymol was 4 mg/mL. They showed a biphasic effect: higher concentrations significantly inhibited planktonic growth (36–58% reduction; *p* < 0.05), while lower concentrations promoted it (10–17% increase; *p* < 0.05). Biofilm biomass varied, but the biofilm index indicated promotion at higher concentrations (0.125–0.5 mg/mL; *p* < 0.05). Both inhibited swarming at 0.5 mg/mL (thymol was more effective). Thymol decreased proteolytic activity, while EO increased pyocyanin production. **Conclusions**: *S. montana* essential oil and thymol show concentration-dependent modulation of *P. aeruginosa* growth, biofilms and virulence, suggesting their potential as anti-virulence agents, although the biphasic responses require careful dosing.

## 1. Introduction

Antimicrobial resistance is one of the biggest public health challenges in the world today. The excessive and irrational use of antibiotics has led to the emergence and spread of multidrug-resistant strains of bacteria, which compromise the effectiveness of existing therapies and increase the mortality rate from infections. In its 2021 report, the World Health Organization points to the worrying stagnation in the development of new antimicrobial agents [1]. In addition, studies from different countries and host species report an ever-increasing prevalence of antimicrobial resistance, highlighting the urgent need to develop global strategies to combat this threat [2,3,4].

The increasing antimicrobial resistance is leading to an intensive search for new therapeutic approaches that could replace or enhance the effectiveness of conventional antibiotics. Natural products, especially plant secondary metabolites, represent a rich and diverse source of bioactive substances that exhibit potent activity against various bacterial strains, including those resistant to multiple drugs [5,6,7,8].

Among plant products, essential oils are one of the most diverse classes of specialised metabolites with a broad spectrum of antimicrobial activities. Their multicomponent composition targets different bacterial structures simultaneously, making resistance less likely than with conventional antibiotics [9,10]. Essential oils and their constituents act as active agents that primarily affect the integrity and function of bacterial membranes, leading to leakage of cell contents and coagulation of the cytoplasm. They also inhibit bacterial efflux pumps, metabolic pathways, and quorum sensing (QS) [11,12]. Essential oils rich in phenolic monoterpenes and phenylpropanes (e.g., thymol, carvacrol, eugenol, cinnamaldehyde) offer broad antibacterial activity, low toxicity, and efficacy against multiple bacteria [13]. One of the plants whose essential oils show promising antimicrobial properties is *Satureja montana* L. (Lamiaceae), commonly known as winter savory or mountain savory. It is an aromatic, perennial subshrub, native to warm, dry, sunny and rocky areas in the Mediterranean region of Southern Europe and North Africa [14,15]. It is valued in traditional medicine for the treatment of gastrointestinal and respiratory diseases, and it is also used in the food and perfume industries [16]. Winter savory produces an essential oil whose profile is dominated by monoterpenes such as thymol, carvacrol, *p*-cymene, geraniol, γ-terpinene, myrcene and linalool. Strong differences in the content of these constituents have been found, confirming the existence of several plant chemotypes [15,17,18,19]. Previous studies showed a strong antimicrobial effect of *S. montana* essential oil against various human [20,21,22], animal [23], plant [24], and foodborne pathogens [25]. It also reduces biofilm production of *Escherichia coli* strains [26], exhibits antibiofilm and anti-adhesive properties against a foodborne *Salmonella* strain [27], and is effective against planktonic and biofilm-embedded cells of *Fusobacterium nucleatum* [28].

*Pseudomonas aeruginosa* is a Gram-negative, motile bacterium that is commonly found in a variety of environments, including moist habitats like water and soil, dry settings, and man-made areas such as hospital facilities, sinks, and ventilators. This versatility enables effective persistence and spread in natural and clinical ecosystems [29]. As an opportunistic pathogen, it causes serious healthcare-associated infections, especially in immunocompromised individuals and those with chronic diseases [30]. Approximately 8–20% of hospitalised patients are colonised or infected with *P. aeruginosa*, with higher rates in vulnerable groups, such as post-surgical patients and those with skin/soft tissue infections (e.g., burns or pressure ulcers), COPD, or cystic fibrosis [31,32,33,34,35]. *P. aeruginosa* contributes to high morbidity and mortality due to its propensity to develop antibiotic resistance, and the World Health Organization has declared it a priority pathogen for which new antimicrobial agents urgently need to be developed [1]. Its adaptability stems from versatile metabolism, inherent and acquired antimicrobial resistance (via efflux pumps, beta-lactamases, and mutations), protective biofilm formation, and diverse virulence factors [30,36]. Biofilm formation, a key survival strategy, is regulated by quorum sensing, which coordinates gene expression and boosts community resilience [37]. Consequently, biofilm cells exhibit greater antibiotic resistance than planktonic cells, which complicates therapy [38], and leads to persistent infections in cystic fibrosis patients or on medical devices.

Complementing its biofilm capabilities, prominent virulence factors of *P. aeruginosa* include cell-surface elements like lipopolysaccharides (for triggering inflammation), pili (for adherence), and flagella (for motility), as well as secreted agents such as elastases and proteases (for tissue degradation), pyocyanin (for inducing oxidative stress), extracellular polysaccharides (for biofilm architecture), and exotoxins (for disrupting cellular functions) [39]. These factors enable severe infections, highlighting the need for therapies that target both virulence and resistance mechanisms.

Essential oils from medicinal plants have shown their potential as antimicrobial and antibiofilm agents against multidrug-resistant bacteria such as *P. aeruginosa*, often targeting planktonic cells, mature biofilms, motility, and virulence factors such as pyocyanin production and quorum sensing [40,41,42,43,44].

Their multicomponent nature promotes synergistic effects and reduces the likelihood of resistance compared to conventional antibiotics. *S. montana* essential oil, rich in the phenolic monoterpenes, exhibits variable antimicrobial activity against *P. aeruginosa* depending on chemotype, origin, and extraction method, with limited to moderate inhibition of planktonic and biofilm forms [26,45,46,47,48]. Thymol, a key constituent, shows biofilm-disrupting potential on surfaces and devices, often in combination with other agents [49,50,51].

Despite these advances, there is limited research addressing the modulating effects of *S. montana* essential oil on biofilm formation and virulence factors of *P. aeruginosa*. Accordingly, our study aimed to address this gap by investigating the antimicrobial, antibiofilm and anti-virulence properties of a thymol-rich chemotype of *S. montana* essential oil and its major constituent, thymol, against the reference strain *P. aeruginosa* PAO1. In particular, we investigated the effects on planktonic growth, biofilm formation, swarm motility and proteolytic activity. Our results could provide valuable insights into innovative plant-based strategies to combat multidrug-resistant *P. aeruginosa* infections, thereby improving clinical therapeutic options and paving the way for optimised formulations of *S. montana* essential oil in anti-biofilm applications.

## 2. Results

### 2.1. Content and Composition of Essential Oil from Winter Savory

The reddish-brown essential oil was isolated from the aerial parts of *S. montana* by hydrodistillation for three hours. The yield of the essential oil was 1.15% (V/m). According to the results of GC-MS analysis, 29 different compounds were identified, which accounted for 99.19% of the total oil content (Table 1). The essential oil was characterised by a very high content of monoterpenes (92.24%), which were predominantly present in oxidized form (75.42%). Sesquiterpenes were only weakly represented (6.65%). The most abundant constituent was thymol (56.47%), followed by thymol methyl ether (8.75%), *p*-cymene (7.24%), carvacrol methyl ether (6.80%) and γ-terpinene (5.77%).

### 2.2. Effect on the Planktonic Growth and Biofilm Formation

In addition to *S. montana* essential oil, we also tested thymol, as its predominant component, with a relative percentage of 56.47. The MICs were 4 mg/mL for both the essential oil and thymol and 2 µg/mL for tobramycin, which was used as a positive control. Tobramycin completely inhibited planktonic growth and biofilm formation at its MIC. Subinhibitory concentrations of essential oil and thymol showed a dual, concentration-dependent effect—both inhibitory and promoting—on bacterial proliferation and biofilm development, which is consistent with previous studies showing that such concentrations of essential oils and their constituents can affect bacterial adhesion and matrix production [46].

**Figure 1:**
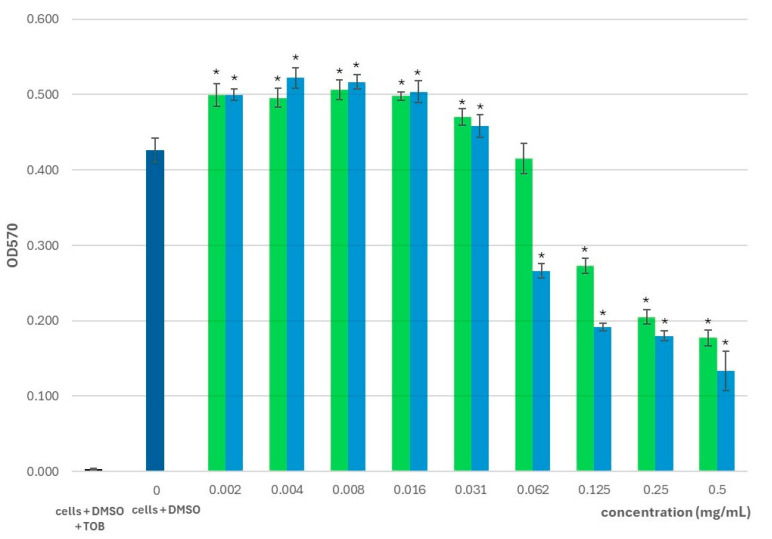
The effect of *S. montana* essential oil (green) and thymol (blue) on the growth of *P. aeruginosa* PAO1 planktonic cells compared to control cells treated with 1% DMSO; TOB—tobramycin; *—concentrations showing significant reduction in the biofilm formation in comparison to the control cells with DMSO (1-ANOVA, *p* < 0.05).

As shown in Figure 1, we observed a significant inhibition of planktonic cell growth for *S. montana* essential oil at higher concentrations (0.125–0.5 mg/mL), with reductions ranging from 36% ± 3% to 58% ± 2% compared to untreated controls. This inhibitory effect diminished as we lowered the concentration, and at 0.062 mg/mL the reduction in optical density was not statistically significant. At lower concentrations (0.002–0.031 mg/mL), a promoting effect was observed, with planktonic growth increasing by 10% ± 3% to 17% ± 4% compared to the control group.

Similarly, thymol exhibited statistically significant inhibitory effects on planktonic growth at all concentrations tested. At higher concentrations (0.062–0.5 mg/mL), a reduction in planktonic growth from 38% ± 2% to 69% ± 6% was observed. The inhibitory effect decreased with decreasing concentration. At lower concentrations (0.002–0.031 mg/mL), thymol stimulated planktonic growth, which increased from 8% ± 4% to 23% ± 3%.

**Figure 2:**
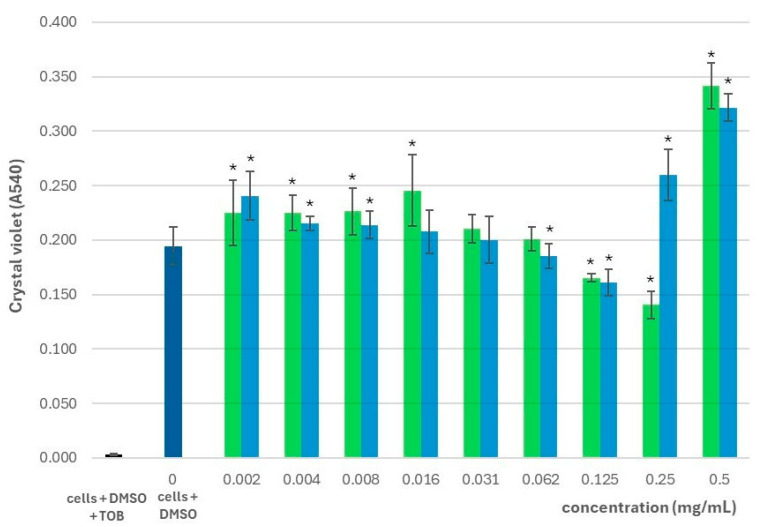
The effect of *S. montana* essential oil (green) and thymol (blue) on the biofilm formation of *P. aeruginosa* PAO1 cells compared to control cells treated with 1% DMSO represented as A_540_ upon staining the biofilm with crystal violet; TOB—tobramycin; *—concentrations showing significant result in comparison to the control cells with DMSO (1-ANOVA, *p* < 0.05).

**Figure 3:**
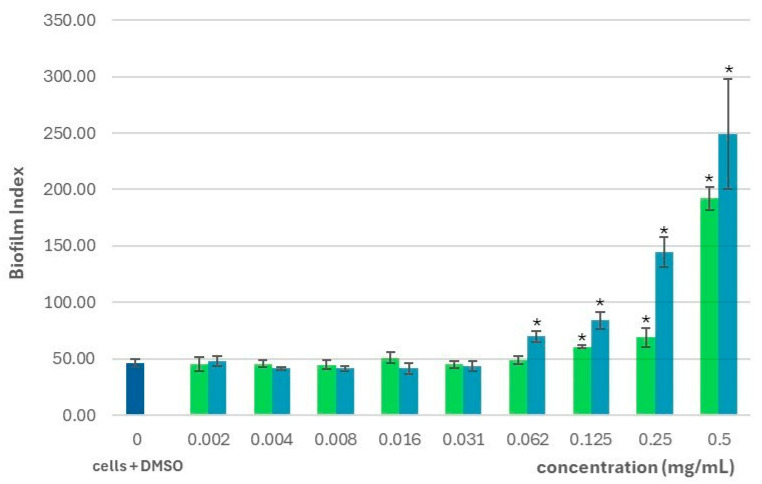
The effect of *S. montana* essential oil (green) and thymol (blue) on the biofilm formation of *P. aeruginosa* PAO1 cells compared to control cells treated with 1% DMSO represented as biofilm index expressed as a ratio (A_540_/A_570_) × 100; *—concentrations showing significant result in comparison to the control cells with DMSO (1-ANOVA, *p* < 0.05).

In terms of biofilm biomass, *S. montana* essential oil significantly decreased biofilm quantity at concentrations of 0.125 and 0.25 mg/mL compared to untreated controls. At other concentrations, we found that the amount of biofilm was generally higher than in the controls, except at 0.031 and 0.062 mg/mL, where no statistically significant differences were observed (Figure 2). In addition to measuring the biofilm mass, we also determined the biofilm index. The significance of the biofilm index is that it integrates both bacterial growth and the amount of biofilm produced into a single parameter, thus illustrating the relative ability of bacteria to form biofilms. In bacteria treated with *S. montana* essential oil, the biofilm index was significantly increased at concentrations between 0.125 and 0.5 mg/mL, indicating enhanced biofilm formation, which increased with increasing concentration. Although the biofilm indices were lower at concentrations from 0.002 to 0.008 mg/mL and at 0.031 mg/mL, these differences were not statistically significant (Figure 3).

Statistically significant differences in biofilm biomass were observed for thymol compared to controls at all concentrations except 0.016 and 0.031 mg/mL. Apart from 0.062 and 0.125 mg/mL, treatment with thymol generally resulted in increased biofilm formation, with the highest absorbance values measured at 0.25 and 0.5 mg/mL. At lower concentrations (0.002–0.031 mg/mL), we observed a progressive decrease in biofilm biomass with decreasing thymol concentration; however, at 0.016 and 0.031 mg/mL, these reductions were not statistically significant (Figure 2). The biofilm index for thymol-treated bacteria was significantly higher than controls in the 0.062–0.5 mg/mL range, suggesting that thymol promotes biofilm formation in a concentration-dependent manner. At concentrations between 0.004 and 0.031 mg/mL, the biofilm index was slightly lower than in the controls, although these differences were not statistically significant (Figure 3).

### 2.3. Effect on Motility and Virulence Factors

**Figure 4:**
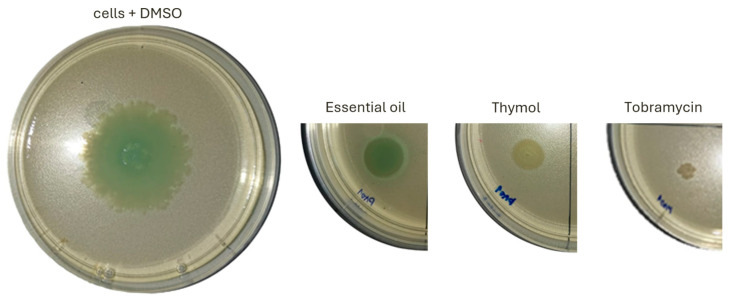
The effect of 0.5 mg/mL *S. montana* essential oil and thymol on the swarming motility of *P. aeruginosa* PAO1 cells compared to control cells treated with 1% DMSO and cells treated with 2 µg/mL tobramycin.

Since the concentration of 0.5 mg/mL of *S. montana* essential oil and thymol was shown to be effective in inhibiting the proliferation of planktonic *P. aeruginosa* cells, we further examined the effects of this concentration on the main virulence factors: swarming motility, proteolytic activity, and pyocyanin production. Both *S. montana* essential oil and thymol inhibited swarming, with thymol showing a stronger inhibitory effect compared to the essential oil (Figure 4). The distance of motility from the application point was measured as follows: control cells (cells + DMSO)—4 cm, cells cultured with essential oil—1.6 cm, and cells cultured with thymol—1.20 cm. Tobramycin, which was used as a positive control at a concentration of 2 μg/mL (MIC in liquid culture), reduced the motility from the application point to 0.5 cm.

**Figure 5:**
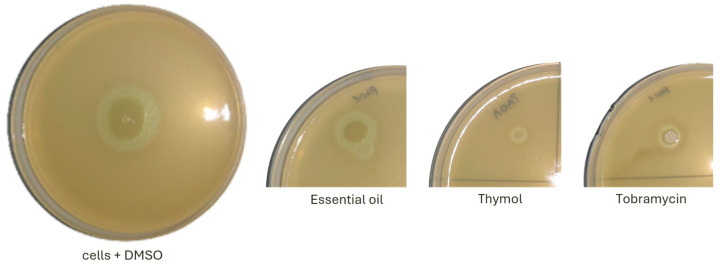
The effect of 0.5 mg/mL *S. montana* essential oil and thymol on the proteolytic activity of *P. aeruginosa* PAO1 cells compared to control cells treated with 1% DMSO and cells treated with 2 µg/mL tobramycin.

To evaluate the proteolytic activity of essential oil and thymol on *P. aeruginosa*, we used a turbid LB medium containing 2% skimmed milk. As Figure 5 shows, the addition of essential oil from *S. montana* had no effect on the proteolytic activity of the PAO1 strain (the radius of the clear zone remained at 6 mm). In contrast, thymol inhibited the proteolytic activity and reduced the radius to 1.5 mm, comparable to tobramycin at concentration of 2 μg/mL (MIC in liquid culture).

The effect of *S. montana* essential oil and thymol on the pyocyanin production of *P. aeruginosa* PAO1 was also investigated. The pyocyanin content was determined by comparing the relative absorbance values of the extracted pyocyanin at 520 nm (A_520_), normalized to the number of bacterial cells in the treated and control culture (cells + DMSO). Tobramycin at concentration of 2 μg/mL completely inhibited bacterial growth. Thymol had no significant effect on pyocyanin production compared to the control (an increase of 4.28% ± 0.3%). Interestingly, the essential oil markedly increased pyocyanin production by 132.70% ± 12.3% compared to the control culture.

## 3. Discussion

Our research has shown that the aerial parts of Croatian winter savory are rich in essential oil (1.15%), which has a high content of monoterpenes, with thymol being the main component (56.5%). Previous studies have shown that the essential oil of *S. montana* exhibits remarkable variability in content and composition due to genetic variability, geographical origin, environmental conditions, harvesting procedures, and extraction method [15,17]. It has been shown that the essential oil content of winter savory can range from 0.22% to 1.75% [17]. Looking at the composition of the essential oil, our sample belongs to the thymol chemotype. The composition is similar to that of a Croatian sample of winter savory, which was also harvested in southern Croatia [52], although carvacrol is even more strongly represented in the phenolic chemotypes [17]. The thymol and carvacrol content of *S. montana* essential oil shows significant geographical differences in the Mediterranean and Balkan regions. For example, the thymol content in Croatian populations can range from a trace to 61% and the carvacrol content from 4.8% to 84%, with both pure and mixed phenolic chemotypes reported [45,52,53,54,55,56]. Similar patterns are observed in Albania, Bosnia and Herzegovina, Italy, Serbia, Portugal, and Spain, where some sites have thymol-rich oils or carvacrol-dominated oils or even considerable contents of other compounds such as linalool and *p*-cymene [15,17,27,48,49,57,58,59].This chemotypic variability emphasises the importance of origin in the selection of plant material for antimicrobial or biofilm applications. Understanding these differences is crucial for optimising the biological activity of *S. montana* essential oil in specific applications.

The essential oils of different *Satureja* species exhibit varying degrees of antimicrobial activity, which depend primarily on their chemical profiles. Among these, *S. montana* is considered one of the most effective [60,61]. The essential oil of *S. montana* L. has demonstrated antimicrobial effects against both Gram-positive and Gram-negative bacteria, including *Staphylococcus aureus*, *Micrococcus luteus, Bacillus subtilis, Listeria monocytogenes, Escherichia coli, Pseudomonas aeruginosa, and Klebsiella pneumoniae* [20,61], as well as fungi such as *Candida albicans* [21,45]. Much of these effects are attributed to the most abundant phenolic monoterpenes in the essential oil, thymol and carvacrol, which are structural isomers, both known for their proven antibacterial properties [13,45,62,63].

Marchese et al. have summarised the antimicrobial effects of thymol against a broad spectrum of pathogenic microorganisms in a systematic literature review [64]. While the antibacterial properties of thymol have long been described, fewer studies have focussed on the effect of thymol upon biofilms, motility and virulence factors. However, several studies have shown that thymol, a major component of the essential oil tested, can significantly suppress biofilms of *Pseudomonas aeruginosa*. One study showed that thymol at a concentration of 0.5 mg/mL significantly reduced the amount of biofilm of *P. aeruginosa* on technical surfaces such as polyvinyl chloride (PVC), polypropylene (PP), polyethylene (PE) and stainless steel (SS) by 70–77% after three days and by 80–100% after ten days, while at the same time reducing the hydrolytic activity of the biofilm by up to 40–100%, with the effect being stronger on PVC and SS than on PE and PP [51]. The results of another study indicated that thymol is an effective inhibitor of biofilms formed by ciprofloxacin-resistant *Pseudomonas aeruginosa* on tympanostomy tubes, with MIC values of 1.56 mg/mL and MBEC values of 3.13–6.25 mg/mL, significantly reducing adhesion by up to 80–90%, biofilm formation by 70–85% and extracellular matrix by 60–95% in a concentration-dependent manner [65]. The most recent study investigated the antimicrobial effect of the combination of thymol and slightly acidic electrolyzed water (SAEW) combination and the eradication of the mature *P. aeruginosa* biofilm. The results indicated that this combination successfully disrupted mature biofilms and significantly reduced the bacterial load of medical catheters [66]. The promising antibiofilm effects of thymol observed in these studies suggest that it may be effective against *P. aeruginosa* not only in planktonic form but also within established biofilms and on various technical surfaces. Building on these findings, we sought to further explore the modulatory effects of both *S. montana* essential oil and its major constituent thymol on *P. aeruginosa* biofilm formation, particularly in relation to their effects on planktonic growth.

In our study, we first determined the minimum inhibitory concentration (MIC) of both *S. montana* essential oil and thymol, which was 4 mg/mL. We then tested the effects of their subinhibitory concentrations ranging from 0.002 to 0.5 mg/mL. This concentration range was chosen to explore the potential antibiofilm and anti-adhesive activities without achieving a complete bactericidal effect. Sub-MIC levels have been shown to decrease bacterial adhesion and matrix production and increase cell membrane permeability, thereby disrupting important physiological processes involved in biofilm development [46]. Previous studies have shown that the antibiofilm and antibacterial activity of *S. montana* essential oil against *P. aeruginosa* is largely dependent on the chemotype of the oil. For example, essential oils with high concentrations of carvacrol often exhibited limited inhibition (MIC and MBC > 4%), illustrating the strong resistance of the pathogen, even to oils rich in phenolic compounds [45]. Essential oils containing high amounts of thymol showed similarly limited activity [48]. On the other hand, some chemotypes with a more balanced or distinct composition, such as those from Bosnia and Herzegovina or from Italy and Albania, showed moderate inhibition with MIC values between 1.56 and 3.12 mg/mL, which can be further improved by formulation as nanoemulsions [26,49]. Extraction methods can also influence the activity, as was found with Serbian oils where microwave extraction increased the inhibitory effect compared to conventional extraction [50]. Overall, these studies emphasise the importance of chemotype and formulation in determining the antibiofilm and antimicrobial activity of *S. montana* essential oil and its constituents against resistant pathogens such as *P. aeruginosa*.

Of particular interest was the observation that the essential oil of *S. montana* exerted both inhibitory and promoting effects on the growth of planktonic cells within the tested concentration range, depending on the concentration applied. At higher concentrations (0.125–0.5 mg/mL), it inhibited the growth of planktonic cells, while at lower concentrations it stimulated growth. When investigating biofilm formation by crystal violet staining alone, the same higher concentrations that inhibited planktonic growth appeared to promote biofilm formation. However, measurement with the biofilm index, which normalises biofilm mass compared to bacterial growth, showed that the actual effect of biofilm formation was less clear than originally thought. This suggests that the apparently increased crystal violet results can be due to differences in biomass or extracellular matrix composition rather than an actual increase in the number of cells forming a biofilm. The lower concentrations of the compounds did not show a significant effect on biofilm formation in any of the measurements. Niu and Gilbert reported similar behaviour in *P. aeruginosa* PAO1 in response to cinnamaldehyde—biofilm production initially decreased but then increased at higher concentrations [67]. This is thought to be due to activation of a stress-induced response and possibly increased production of exopolysaccharides, ultimately leading to stronger crystal violet binding [68,69].

A dual effect on the growth of planktonic cells was also observed with thymol, which has an inhibitory effect on planktonic growth at higher concentrations and a growth-promoting effect at lower concentrations, with the inhibitory concentration range being somewhat broader (0.062–0.5 mg/mL). Similarly to *S. montana* essential oil, thymol promoted biofilm formation at the same concentrations that inhibit planktonic growth. Considering that thymol is one of the most abundant components of *S. montana* L. essential oil, such an effect of thymol would have been expected. However, a comparison of the obtained values revealed that both the inhibitory effect on bacterial growth and the promoting effect on biofilm formation were more pronounced in the case of thymol. This could be due to the fact that the essential oil of *S. montana* L. contains numerous other components, in addition to thymol, which are probably involved in complex interactions. These interactions may be additive, synergistic or antagonistic and ultimately determine the effect of the essential oil itself, which may differ considerably from the effects of the individual constituents [10,70]. More recent studies provide additional support for our findings. Liu et al. demonstrated that thymol has a concentration-dependent dual effect on *P. aeruginosa* biofilms, with low concentrations promoting biofilm formation and higher concentrations inhibiting it [71]. Similar biphasic or paradoxical responses have been reported for other essential oils and phenolic compounds, such as cinnamaldehyde [67], suggesting a mechanism of stress adaptation possibly mediated by increased EPS production or altered cell signalling. These observations emphasise that the antibiofilm activity of thymol and thus also of essential oil from *S. montana* may be independent or even opposite to its antibacterial effect against planktonic cells in certain concentration ranges. In view of these opposing effects on biofilm formation and planktonic growth, the antibiofilm activity of *S. montana* essential oil and thymol could therefore be independent of their antibacterial activity.

In addition to their effects on planktonic growth and biofilm formation, our results show that *S. montana* essential oil and thymol can modulate key virulence factors of *P. aeruginosa*, namely swarming motility and proteolytic activity. Swarming motility, which is under the control of the quorum sensing mechanism and contributes to significant surface colonisation and biofilm production, was inhibited by both treatments at 0.5 mg/mL, with thymol showing a stronger effect (reducing the motility distance from 4 cm in the control to 1.2 cm). While the essential oil of *S. montana* did not markedly affect the proteolytic activity of *P. aeruginosa*, thymol significantly inhibited protease production, as shown by the marked reduction of the clear zone in the skimmed milk agar assay. These results suggest that both the essential oil and its major constituent, thymol, may attenuate key virulence mechanisms beyond the direct effects on bacterial growth and biofilm biomass, with thymol alone possibly having a stronger effect. Based on these observations, our results are consistent with previous studies on essential oils and their phenolic constituents against *P. aeruginosa*. For example, *Plectranthus barbatus* essential oil (2.5% *v*/*v*) inhibited swarming and twitching motility of *P. aeruginosa* PAO1 by downregulating QS-dependent genes [72], while the essential oils of *Origanum vulgare* and *Coridothymus capitatus* reduced swarming by interfering with QS [73,74]. Similarly, *Illicium verum* essential oil inhibited swarming by 38% at 100 µg/mL [75] and *Cuminum cyminum* essential oil and cuminaldehyde reduced motility by up to 90% at 500 mg/mL, while suppressing QS-regulated virulence enzymes such as elastase (62–63%) and protease (82–83%) [76]. Other essential oils, e.g., from caraway (63.7% carvone), reduced biofilm formation by 60–72% and suppressed virulence but risked residual pathogenicity without eradication [77]. *Eucalyptus* essential oil inhibited initial adhesion (58–95%) at 10–20 µL/mL [78].

With regard to pyocyanin production, a prominent quorum sensing (QS)-controlled virulence factor of *Pseudomonas aeruginosa* associated with oxidative stress and tissue damage, our results showed contrasting effects between *S. montana* essential oil and its major component thymol. At a concentration of 0.5 mg/mL, thymol showed no significant effect, but only a marginal increase of 4.28% ± 0.3% compared to controls, indicating limited interference with pyocyanin biosynthetic pathways. In contrast, the essential oil significantly enhanced pyocyanin production by 132.70% ± 12.3%, potentially indicating a stimulatory effect on QS signalling or associated metabolic processes, possibly due to synergistic interactions between its multicomponent profiles beyond thymol alone. These results are in contrast to recent studies on other essential oils, where QS-regulated pyocyanin is generally inhibited. For example, *Ocimum basilicum* essential oil reduced pyocyanin levels by 13.3–55.6%, while *Salvia officinalis* essential oil achieved inhibition of up to 58.7% at sub-MICs (MIC 5–20 mg/mL) in several *P. aeruginosa* strains tested, effects attributed to QS disruption [41]. Similarly, *Coridothymus capitatus* essential oil almost eliminated pyocyanin synthesis in 11 of 12 tested *P. aeruginosa* strains, with near-complete inhibition in some isolates, which is also associated with QS interference [74]. These discrepancies could be due to chemotypical variations in *S. montana* oil, concentration-dependent responses, or unique interactions between constituents that promote rather than suppress virulence under certain conditions, highlighting the need for further mechanistic studies to elucidate these biphasic modulatory effects and optimise therapeutic applications.

In summary, our study has shown that the thymol-rich essential oil of *S. montana* has a concentration-dependent dual effect on both planktonic growth and biofilm development of *P. aeruginosa* and modulates two important virulence factors, namely swarming motility and proteolytic activity. Our data, which are consistent with previous findings on phenol-rich essential oils, emphasise the potential of *S. montana* essential oil as a multifunctional anti-virulence therapeutic agent against multidrug-resistant pathogens. By underscoring the chemotypic variability and non-linear responses, this study highlights the need for optimised harvesting, formulation and dosing procedures to effectively utilise the essential oils.

This study demonstrates the pharmaceutical potential of *S. montana* essential oil as a promising candidate for the development of new therapeutic approaches against multidrug-resistant *P. aeruginosa*. The proven antimicrobial, antibiofilm, and antiviral activities of this thymol-rich essential oil support its potential application in topical wound healing and inhalation therapy for respiratory tract infections, including those associated with cystic fibrosis. The results presented emphasise the value of plant-derived antimicrobials as a safe and effective adjunct to conventional antibiotics and underscore the need for future in vivo and clinical studies with *S. montana* essential oil, targeting biofilm-associated infections.

## 4. Materials and Methods

### 4.1. Plant Material

The aerial parts of wild-growing *Satureja montana* L. were harvested at the full flowering stage in July 2022 from South Velebit Mt., Croatia (44°19′30.4″ N 15°26′57.3″ E). The plant was authenticated by the Department of Pharmacognosy, Faculty of Pharmacy and Biochemistry (University of Zagreb, Croatia), where the voucher specimen was also deposited under the genus number 819. The collected plant material was air-dried in the shade at room temperature.

### 4.2. Isolation of Essential Oil

The essential oil of *S. montana* was isolated by hydrodistillation according to the European Pharmacopoeia [79]. The dried plant material was crushed and distilled in a Clevenger-type apparatus for 3 h at a rate of 2–3 mL/min. The yield (%, V/m) was calculated as the volume of essential oil (mL) per 100 g of plant material. The collected essential oil was dried with anhydrous sodium sulphate and stored in a refrigerator at 4 °C until further analysis.

### 4.3. Analysis of Essential Oil by Gas Chromatography-Mass Spectrometry (GC-MS)

The components were separated on a non-polar Agilent Technologies (Santa Clara, CA, USA) HP-5 MS capillary column (30 m × 0.25 mm, film thickness 0.25 μm). The following temperature programme was used: isothermal at 60 °C for 1 min, increasing from 60 °C to 200 °C at a rate of 3 °C/min and holding isothermal at 200 °C for 10 min. Mass spectra were recorded at 70 eV and scanned in the range 40–400 *m*/*z*. Data were acquired and processed using Agilent GC/MS ChemStation software E.02. The components were identified by comparing their mass spectra with the spectra stored in the NIST 2020 or reported in the literature. Identification was also performed by comparing their retention indices (RI) with the values reported in the literature [80]. The linear retention indices were determined considering a homologous series of n-alkanes (C8–C24) analysed under the same operating conditions. The relative amounts of the components, expressed as percentages, were calculated by a normalisation procedure based on the peak area in the total ion chromatogram.

### 4.4. Determination of Minimum Inhibitory Concentration (MIC)

The minimum inhibitory concentration (MIC) for *Pseudomonas aeruginosa* PAO1 (Leibnitz Institute DSMZ 1707, Braunschweig, Germany) was determined for the essential oil of *Satureja montana* L., thymol and tobramycin as a positive control in M63 medium supplemented with 0.4% Arg using the broth microdilution method [81]. *P. aeruginosa* cells were grown overnight in Luria–Bertani (LB) medium and then diluted to an optical density of 0.5 McFarland (10^8^ CFU/mL) in fresh LB medium. Cells were further diluted 1:100 with a serial dilution of essential oil or thymol in M63 medium containing 0.4% Arg, and 100 µL aliquots (10^5^ CFU) were spread on into 96-well plates. The concentration range tested for essential oil and thymol (initially dissolved in DMSO at a concentration of 100 mg/mL) was 0.5–4 mg/mL (0.5 mg/mL, 1 mg/mL, 2 mg/mL and 4 mg/mL) and for tobramycin, it was 0.5–4 µg/mL (0.5 µg/mL, 1 µg/mL, 2 µg/mL and 4 µg/mL). After incubation at 37 °C for 24 h, the MIC was determined by measuring the cell density at 570 nm (Wallac Victor2 1420, Perkin Elmer, Shelton, CT, USA) to indicate the minimum concentration that prevents visible growth. The MIC was determined in three independent experiments for each of the substances examined.

The concentrations of essential oil and thymol for the subsequent assays were selected based on the preliminary MIC determinations (4 mg/mL for both the essential oil and thymol). Subinhibitory concentrations ranging from 0.002 to 0.5 mg/mL were then tested to evaluate modulatory effects on biofilm formation, motility, and virulence factors without achieving a complete bactericidal effect. This approach builds on established protocols in similar studies on essential oils against *P. aeruginosa* and ensures the assessment of non-lethal, biologically relevant doses, taking into account the volatility and complexity of the essential oil [44,66,71].

### 4.5. Anti-Biofilm Assay

The effect of *Satureja montana* L. essential oil and thymol on biofilm formation of *Pseudomonas aeruginosa* PAO1 was investigated using a modified method by Vitanza et al. [20]. We performed three independent experiments with 8 technical replicates per experiment. *P. aeruginosa* was grown overnight in Luria–Bertani (LB) medium and diluted with LB medium to an optical density of 0.5 McFarland. To initiate QS-dependent biofilm formation, we performed an additional dilution step at a ratio 1:100 to achieve an optical density of approximately 0.01 at 600 nm. In this second step, the bacterial culture was diluted with 1% DMSO in M63 medium with 0.4% Arg (control) or with the M63 medium containing 0.4% Arg and essential oil or thymol at concentrations of 0.002 mg/mL, 0.004 mg/mL, 0.008 mg/mL, 0.016 mg/mL, 0.031 mg/mL, 0.062 mg/mL, 0.125 mg/mL, 0.25 mg/mL and 0.5 mg/mL (stock solutions of essential oil and thymol were 100 mg/mL in DMSO). Tobramycin was used as a positive control at a MIC of 2 μg/mL. One hundred µL were transferred to the 96-well U-bottom microtiter plate in eight technical replicates. The plates were incubated aerobically at 37 °C for 24 h. The biofilm was detected as described in [82]. Bacterial growth of the planktonic cells was measured at 570 nm (Wallace Victor 2 1420, Perkin Elmer). Planktonic cells were removed and biofilm was stained with 125 µL of 0.1% crystal violet for 15 min. The microtiter plate was rinsed three times with H_2_O, dried and biofilm extracted with 150 µL of 30% acetic acid. An amount of 125 µL of the dissolved crystal violet was transferred to the new flat-bottom microtiter plate and the absorbance was measured at 540 nm (Wallac Victor 2 1420, Perkin Elmer). The biofilm index, which represents the amount of developed biofilm related to bacterial growth [67], was calculated as a ratio (A_540_/A_570_) × 100 [83].

### 4.6. Swarming Motility Assay

Swarming, an important virulence factor regulated by the quorum sensing system of *P. aeruginosa*, represents coordinated bacterial movement on semi-solid media such as LB medium containing 0.5% agar, as used in this study. Under these conditions, the bacteria typically show dendritic swarming patterns with multi-branched layers [47]. The swarming motility of the bacterial strains *Pseudomonas aeruginosa* PAO1 was tested on semi-solid LB-medium containing 0.5% agar with the addition of the essential oil of *Satureja montana* L. or thymol at concentrations of 0.5 mg/mL. Tobramycin was used as a positive control at a MIC of 2 μg/mL. The bacterial cells were grown aerobically overnight in liquid LB medium. An aliquot of 1.5 μL of the overnight culture was applied to the centre of the LB plate and the plates were incubated at 37 °C for 24 h [84]. The results were analysed by visual inspection and by measuring the motility distance from the application point. The assay was performed in three independent experiments.

### 4.7. Proteolytic Activity Assay

*P. aeruginosa* secretes various proteases that degrade casein in the milk, resulting in a clear zone around the bacterial colony. The addition of test substances to the medium can alter the proteolytic activity, which is reflected in the changes in the radius of the clear zone around the bacterial cells [84]. To evaluate proteolytic activity, we applied a semi-quantitative method using turbid LB medium with 2% skimmed milk with the addition of the *Satureja montana* L. essential oil or thymol in concentrations of 0.5 mg/mL. Tobramycin was used as a positive control at a MIC of 2 μg/mL. Bacterial cells were grown overnight aerobically in liquid LB medium. The results were analysed by visual inspection and by measuring the motility distance from the application point. The assay was performed in three independent experiments.

### 4.8. Pyocyanin Production Assay

*Pseudomonas aeruginosa* PAO1 cells were grown overnight in LB medium, adjusted to 0.5 McFarland with LB medium, and diluted 1:100 in 5 mL LB medium containing either 1% DMSO (control), 0.5 mg/mL essential oil or 0.5 mg/mL thymol. Tobramycin was used as a positive control at a MIC of 2 μg/mL. The culture was grown aerobically at 37 °C for 24 h and bacterial growth was measured at A_570_. Pyocyanin was extracted as described previously [85]. The cells were pelleted and the pyocyanin was extracted from the supernatant with 3 mL of chloroform, centrifuged at 5000 rpm for 10 min (Thermo Jouan BR4i, Walthamm, MA, USA) and the chloroform layer was re-extracted with 1 mL of 0.2 M HCl. The absorbance was measured at 520 nm, and the results were expressed as the ratio (A_520_/A_570_) × 100 to normalise the pyocyanin levels to cell growth. Three independent experiments were performed with two technical replicates per experiment.

### 4.9. Statistical Analysis

For the results obtained in antibiofilm and pyocyanin production assays, statistical significance was determined using one-way ANOVA, with *p*-values < 0.05 considered significant.

## 5. Conclusions

### 5.1. Key Findings

The results of our study showed that both the thymol-rich essential oil of *S. montana* and its main constituent thymol have complex, concentration-dependent modulatory effects on *Pseudomonas aeruginosa* PAO1. In particular, at higher subinhibitory concentrations, they inhibit planktonic growth and swarming motility but paradoxically promote biofilm formation; the essential oil also enhances pyocyanin production, while thymol suppresses proteolytic activity. This emphasises the potentially superior anti-virulence profile of thymol, which is probably due to synergistic or antagonistic interactions between oil components. In particular, our results for pyocyanin, a prominent quorum sensing-regulated virulence factor associated with oxidative stress and tissue damage, showed striking differences: thymol had a negligible effect, while the essential oil significantly increased pyocyanin production, likely through stimulatory effects on QS or metabolic pathways driven by its multicomponent synergies.

### 5.2. Implications and Future Directions

Overall, *S. montana* essential is a promising natural adjunct to conventional antibiotics in the control of multidrug-resistant *P. aeruginosa* infections, particularly in biofilm-associated infections such as cystic fibrosis or medical device-related infections. Future research should investigate in vivo efficacy, synergistic combinations with existing antimicrobial agents, and clinical trials to validate these effects and address potential limitations such as chemotype variability and non-linear dose responses. Such investigations could elucidate the mechanisms behind these modulatory discrepancies and enable customised, plant-based therapies that effectively target virulence and quorum sensing without unintended amplification. By integrating plant-based strategies, we can develop innovative therapies to combat antimicrobial resistance.

## Figures and Tables

**Table 1:** Composition of the hydrodistilled essential oil of *Satureja montana* L.

No. ^a^	RI ^b^	Compound	Relative Percentage (%)
1	925	α-Thujene	0.53
2	932	α-Pinene	0.34
3	947	Camphene	0.17
4	977	1-Octen-3-ol	0.30
5	990	Myrcene	0.60
6	1005	α-Phellandrene	0.14
7	1016	α-Terpinene	1.43
8	1023	*p*-Cymene	7.24
9	1027	Limonene	0.31
10	1036	*cis*-β-Ocimene	0.29
11	1057	γ-Terpinene	5.77
12	1066	*cis*-Sabinene hydrate	0.12
13	1101	Linalool	0.17
14	1164	Borneol	0.83
15	1176	Terpinen-4-ol	0.66
16	1234	Thymol methyl ether	8.75
17	1239	Pulegone	0.22
18	1243	Carvacrol methyl ether	6.80
19	1294	Thymol	56.47
20	1303	Carvacrol	1.18
21	1354	Thymol acetate	0.22
22	1417	β-Caryophyllene	3.10
23	1474	γ-Muurolene	0.14
24	1492	γ-Amorphene	0.19
25	1507	β-Bisabolene	0.35
26	1511	γ-Cadinene	0.16
27	1522	δ-Cadinene	0.29
28	1574	Spathulenol	0.12
29	1580	Caryophyllene oxide	2.30

^a^ Compounds are listed according to their elution from a HP-5MS column. ^b^ Retention index on HP-5 MS column.

## Data Availability

The data presented in this study are available in this article.

## References

[1] WHO (2022). 2021 Antibacterial Agents in Clinical and Preclinical Development.

[2] Salam M.A., Al-Amin M.Y., Salam M.T., Pawar J.S., Akhter N., Rabaan A.A., Alqumber M.A.A. (2023). Antimicrobial Resistance: A Growing Serious Threat for Global Public Health. Healthcare.

[3] Naghavi M., Vollset S.E., Ikuta K.S., Swetschinski L.R., Gray A.P., Wool E.E., Aguilar G.R., Mestrovic T., Smith G., Han C. (2024). Global Burden of Bacterial Atimicrobial Resistance 1990–2021: A Systematic Analysis with Forecasts to 2050. Lancet.

[4] Javed M.U., Ijaz M., Ahmed A., Rasheed H., Sabir M., Jabir A. (2024). Molecular Dynamics and Antimicrobial Resistance Pattern of β-lactam Resistant Coagulase Positive *Staphylococcus aureus* Isolated from Goat Mastitis. PVJ.

[5] Salikin N.H., Keong L.C., Azemin W.-A., Philip N., Yusuf N., Daud S.A., Rashid S.A. (2024). Combating Multidrug-Resistant (MDR) *Staphylococcus aureus* Infection Using Terpene and Its Derivative. World J. Microbiol. Biotechnol..

[6] Aljohani A.S.M. (2023). Botanical Compounds: A Promising Approach to Control Mycobacterium Species of Veterinary and Zoonotic Importance. PVJ.

[7] Ibrahim E., Abdalhamed A., Arafa A., Eid R., Halil H.M., Hedia R., Dorgham S., Hozyen H. (2023). In vitro and in vivo Antibacterial and Antibiofilm Efficacy of Selenium Nanoparticles against *Staphylococcus aureus* Supported with Toxicopathological and Behavioral Studies in Rats. Int. J. Vet. Sci..

[8] Neagu R., Popovici V., Ionescu L.E., Ordeanu V., Popescu D.M., Ozon E.A., Gîrd C.E. (2023). Antibacterial and Antibiofilm Effects of Different Samples of Five Commercially Available Essential Oils. Antibiotics.

[9] Chouhan S., Sharma K., Guleria S. (2017). Antimicrobial Activity of Some Essential Oils—Present Status and Future Perspectives. Medicines.

[10] Bakkali F., Averbeck S., Averbeck D., Idaomar M. (2008). Biological Effects of Essential Oils—A Review. Food Chem. Toxicol..

[11] Reichling J. (2020). Anti-biofilm and Virulence Factor-Reducing Activities of Essential Oils and Oil Components as a Possible Option for Bacterial Infection Control. Planta Med..

[12] Trifan A., Luca S.V., Greige-Gerges H., Miron A., Gille E., Aprotosoaie A.C. (2020). Recent Advances in Tackling Microbial Multidrug Resistance with Essential Oils: Combinatorial and Nano-Based Strategies. Crit. Rev. Microbiol..

[13] Khwaza V., Aderibigbe B.A. (2025). Antibacterial Activity of Selected Essential Oil Components and Their Derivatives: A Review. Antibiotics.

[14] Les F., Galiffa V., Cásedas G., Moliner C., Maggi F., López V., Gómez-Rincón C. (2024). Essential Oils of Two Subspecies of *Satureja montana* L. against Gastrointestinal Parasite *Anisakis simplex* and Acetylcholinesterase Inhibition. Molecules.

[15] Maccelli A., Vitanza L., Imbriano A., Fraschetti C., Filippi A., Goldoni P., Maurizi L., Ammendolia M.G., Crestoni M.E., Fornarini S. (2019). *Satureja montana* L. Essential Oils: Chemical Profiles/Phytochemical Screening, Antimicrobial Activity and O/W NanoEmulsion Formulations. Pharmaceutics.

[16] Said-Al Ahl H.A.H., Kačániova M., Mahmoud A.A., Hikal W.M., Čmiková N., Szczepanek M., Błaszczyk K., Al-Balawi S.M., Bianchi A., Smaoui S. (2024). Phytochemical Characterization and Biological Activities of Essential Oil from *Satureja montana* L., a Medicinal Plant Grown under the Influence of Fertilization and Planting Dates. Biology.

[17] Hajdari A., Mustafa B., Kaçiku A., Mala X., Lukas B., Ibraliu A., Stefkov G., Novak J. (2016). Chemical Composition of the Essential Oil, Total Phenolics, Total Flavonoids and Antioxidant Activity of Methanolic Extracts of *Satureja montana* L.. Rec. Nat. Prod..

[18] Dodoš T., Janković S., Marin P.D., Rajčević N. (2021). Essential Oil Composition and Micromorphological Traits of *Satureja montana* L., *S. subspicata* Bartel ex Vis., and *S. kitaibelii* Wierzb. Ex Heuff. Plant Organs. Plants.

[19] Capdevila S., Grau D., Cristóbal R., Moré E., De Las Heras X. (2025). Chemical Composition of Wild Populations of *Thymus vulgaris* and *Satureja montana* in Central Catalonia, Spain. JSFA Rep..

[20] Vitanza L., Maccelli A., Marazzato M., Scazzocchio F., Comanducci A., Fornarini S., Crestoni M.E., Filippi A., Fraschetti C., Rinaldi F. (2019). *Satureja montana* L. Essential Oil and Its Antimicrobial Activity Alone or in Combination with Gentamicin. Microb. Pathog..

[21] Aćimović M., Stanković Jeremić J., Todosijević M., Cvetković M., Lončar B., Vukić V., Erceg T., Pezo L., Zheljazkov V.D. (2024). The Influence of Weather Conditions on the Immortelle Volatile Constituents from Essential oil and Hydrosol with a Focus on Italidiones and Its Molecular Docking Anti-Inflammatory Potential. Nat. Product. Commun..

[22] Rezende D.A.d.C.S., Oliveira C.D., Batista L.R., Ferreira V.R.F., Brandão R.M., Caetano A.R.S., Alves M.V.P., Cardoso M.G. (2022). Bactericidal and Antioxidant Effects of Essential Oils from *Satureja montana* L., *Myristica fragrans* H., and *Cymbopogon flexuosus*. Lett. Appl. Microbiol..

[23] Ebani V.V., Bertelloni F., Najar B., Nardoni S., Pistelli L., Mancianti F. (2020). Antimicrobial Activity of Essential Oils against *Staphylococcus* and *Malassezia* Strains Isolated from Canine Dermatitis. Microorganisms.

[24] Cagnoli G., Bertelloni F., Ebani V.V. (2024). In Vitro Antibacterial Activity of Essential Oils from *Origanum vulgare*, *Satureja montana*, *Thymus vulgaris*, and Their Blend Against Necrotoxigenic (NTEC), Enteropathogenic (EPEC), and Shiga-Toxin Producing *Escherichia coli* (STEC) Isolates. Pathogens.

[25] Šojić B., Ikonić P., Kocić-Tanackov S., Peulić T., Teslić N., Županjac M., Lončarević I., Zeković Z., Popović M., Vidaković S. (2023). Antibacterial Activity of Selected Essential Oils against Foodborne Pathogens and Their Application in Fresh Turkey Sausages. Antibiotics.

[26] Rinaldi F., Maurizi L., Conte A.L., Marazzato M., Maccelli A., Crestoni M.E., Hanieh P.N., Forte J., Conte M.P., Zagaglia C. (2021). Nanoemulsions of *Satureja montana* Essential Oil: Antimicrobial and Antibiofilm Activity against Avian *Escherichia coli* Strains. Pharmaceutics.

[27] Miladi H., Mili D., Ben Slama R., Zouari S., Ammar E., Bakhrouf A. (2016). Antibiofilm Formation and Anti-Adhesive Property of Three Mediterranean Essential Oils Against a Foodborne Pathogen *Salmonella* Strain. Microbial. Pathogenesis.

[28] Ben Lagha A., Vaillancourt K., Maquera Huacho P., Grenier D. (2020). Effects of Labrador Tea, Peppermint, and Winter Savory Essential Oils on *Fusobacterium nucleatum*. Antibiotics.

[29] Ramos J.-L. (2004). Pseudomonas: Volume 1 Genomics, Life Style and Molecular Architecture.

[30] Qin S., Xiao W., Zhou C., Pu Q., Deng X., Lan L., Liang H., Song X., Wu M. (2022). *Pseudomonas aeruginosa*: Pathogenesis, Virulence Factors, Antibiotic Resistance, Interaction with Host, Technology Advances and Emerging Therapeutics. Signal Transduct. Target. Ther..

[31] Eklöf J., Sørensen R., Ingebrigtsen T.S., Sivapalan P., Achir I., Boel J.B., Bangsborg J., Ostergaard C., Dessau R.B., Jensen U.S. (2020). *Pseudomonas aeruginosa* and Risk of Death and Exacerbations in Patients with Chronic Obstructive Pulmonary Disease: An Observational Cohort Study of 22,053 Patients. Clin. Microbiol. Infect..

[32] Recanatini C., Van Werkhoven C.H., Van Der Schalk T.E., Paling F., Hazard D., Timbermont L., Torrens G., DiGiandomenico A., Esser M.T., Wolkewitz M. (2025). Impact of *Pseudomonas aeruginosa* Carriage on Intensive Care Unit-Acquired Pneumonia: A European Multicentre Prospective Cohort Study. Clin. Microbiol. Infect..

[33] Sanjar F., Millan C.P., Leung K.P. (2024). Phylogenetic Evaluation and Genotypic Identification of Burn-Related *Pseudomonas aeruginosa* Strains Isolated from Post-Burn Human Infections During Hospitalization. Pathog. Dis..

[34] Harris A.D., Jackson S.S., Robinson G., Pineles L., Leekha S., Thom K.A., Wang Y., Doll M., Pettigrew M.M., Johnson J.K. (2016). *Pseudomonas aeruginosa* Colonization in the Intensive Care Unit: Prevalence, Risk Factors, and Clinical Outcomes. Infect. Control Hosp. Epidemiol..

[35] Malhotra S., Hayes D., Wozniak D.J. (2019). Cystic Fibrosis and *Pseudomonas aeruginosa*: The Host-Microbe Interface. Clin. Microbiol. Rev..

[36] Fernández-Billón M., Llambías-Cabot A.E., Jordana-Lluch E., Oliver A., Macià M.D. (2023). Mechanisms of Antibiotic Resistance in *Pseudomonas aeruginosa* Biofilms. Biofilm.

[37] Lee J., Zhang L. (2015). The Hierarchy Quorum Sensing Network in *Pseudomonas aeruginosa*. Protein Cell.

[38] Vestby L.K., Grønseth T., Simm R., Nesse L.L. (2020). Bacterial Biofilm and its Role in the Pathogenesis of Disease. Antibiotics.

[39] Jurado-Martín I., Sainz-Mejías M., McClean S. (2021). *Pseudomonas aeruginosa*: An Audacious Pathogen with an Adaptable Arsenal of Virulence Factors. Int. J. Mol. Sci..

[40] Stojanović-Radić Z., Pejčić M., Stojanović N., Sharifi-Rad J., Stanković N. (2016). Potential of *Ocimum basilicum* L. and *Salvia officinalis* L. Essential Oils Against Biofilms of *P. aeruginosa* Clinical Isolates. Cell Mol. Biol..

[41] Pejčić M., Stojanović-Radić Z., Genčić M., Dimitrijević M., Radulović N. (2020). Anti-Virulence Potential of Basil and Sage Essential Oils: Inhibition of Biofilm Formation, Motility and Pyocyanin Production of *Pseudomonas aeruginosa* Isolates. Food Chem. Toxicol..

[42] Haripriyan J., Binu C.R., Menon N.D., Vanuopadath M., Hari M.B., Namitha N., Binoy K., Kumar A., Nair B.G., Nizet V. (2025). Essential Oils Modulate Virulence Phenotypes in a Multidrug-Resistant Pyomelanogenic *Pseudomonas aeruginosa* Clinical Isolate. Sci. Rep..

[43] Coșeriu R.L., Vintilă C., Pribac M., Mare A.D., Ciurea C.N., Togănel R.O., Cighir A., Simion A., Man A. (2023). Antibacterial Effect of 16 Essential Oils and Modulation of mex Efflux Pumps Gene Expression on Multidrug-Resistant *Pseudomonas aeruginosa* Clinical Isolates: Is Cinnamon a Good Fighter?. Antibiotics.

[44] Van L.T., Hagiu I., Popovici A., Marinescu F., Gheorghe I., Curutiu C., Ditu L.M., Holban A.-M., Sesan T.E., Lazar V. (2022). Antimicrobial Efficiency of Some Essential Oils in Antibiotic-Resistant *Pseudomonas aeruginosa* Isolates. Plants.

[45] Skočibušić M., Bezić N. (2004). Chemical Composition and Antimicrobial Variability of *Satureja montana* L. Essential Oils Produced During Ontogenesis. J. Essent. Oil Res..

[46] Čabarkapa I., Čolović R., Đuragić O., Popović S., Kokić B., Milanov D., Pezo L. (2019). Anti-Biofilm Activities of Essential Oils Rich in Carvacrol and Thymol Against *Salmonella enteritidis*. Biofouling.

[47] Kollaran A.M., Joge S., Kotian H.S., Badal D., Prakash D., Mishra A., Varma M., Singh V. (2019). Context-Specific Requirement of Forty-Four Two-Component Loci in *Pseudomonas aeruginosa* Swarming. iScience.

[48] Bezbradica D.I., Tomovic J.M., Vukasinovic M.S., Siler-Marinkovic S., Ristic M.M. (2005). Composition and Antimicrobial Activity of Essential Oil of *Satureja montana* L. Collected in Serbia and Montenegro. J. Essent. Oil Res..

[49] Ćavar S., Maksimović M., Šolić M.E., Jerković-Mujkić A., Bešta R. (2008). Chemical Composition and Antioxidant and Antimicrobial Activity of Two *Satureja* Essential Oils. Food Chem..

[50] Djordjevic N., Mancic S., Karabegovic I., Cvetkovic D., Stanojevic J., Savic D., Danilovic B. (2021). Influence of the Isolation Method to the Composition and Antimicrobial and Antioxidative Activity of Winter Savory (*Satureja montana* L.) Essential Oil. J. Essent. Oil Bear. Plants.

[51] Walczak M., Michalska-Sionkowska M., Olkiewicz D., Tarnawska P., Warżyńska O. (2021). Potential of Carvacrol and Thymol in Reducing Biofilm Formation on Technical Surfaces. Molecules.

[52] Mastelić J., Jerković I. (2003). Gas Chromatography–Mass Spectrometry Analysis of Free and Glycoconjugated Aroma Compounds of Seasonally Collected *Satureja montana* L.. Food Chemistry.

[53] Skočibušić M., Bezić N. (2004). Phytochemical Analysis and In Vitro Antimicrobial Activity of Two *Satureja* Species Essential Oils. Phytother. Res..

[54] Milos M., Radonic A., Bezic N., Dunkic V. (2001). Localities and Seasonal Variations in the Chemical Composition of Essential Oils of *Satureja montana* L. and *S. cuneifolia* Ten. Flavour Fragr. J..

[55] Radonic A., Milos M. (2003). Chemical Composition and In Vitro Evaluation of Antioxidant Effect of Free Volatile Compounds from *Satureja montana* L.. Free Radic. Res..

[56] Stanic G., Petricic J., Blazevic N. (1991). Gas Chromatographic Investigations of Essential Oils of *Satureja montana* and *Satureja subspicata* from Yugoslavia. J. Essent. Oil Res..

[57] Ibraliu A., Dhillon B.S., Faslia N., Stich B. (2010). Variability of Essential Oil Composition in Albanian Accessions of *Satureja montana* L.. J. Med. Plants Res..

[58] Djenane D., Yangüela J., Montañés L., Djerbal M., Roncalés P. (2011). Antimicrobial Activity of *Pistacia lentiscus* and *Satureja montana* Essential Oils Against *Listeria monocytogenes* CECT 935 Using Laboratory Media: Efficacy and Synergistic Potential in Minced Beef. Food Control.

[59] Serrano C., Matos O., Teixeira B., Ramos C., Neng N., Nogueira J., Nunes M.L., Marques A. (2011). Antioxidant and Antimicrobial Activity of *Satureja montana* L. Extracts. J. Sci. Food Agric..

[60] Tepe B., Cilkiz M. (2016). A Pharmacological and Phytochemical Overview on *Satureja*. Pharm. Biol..

[61] Abbad I., Soulaimani B., Iriti M., Barakate M. (2025). Chemical Composition and Synergistic Antimicrobial Effects of Essential Oils From Four Commonly Used Satureja Species in Combination With Two Conventional Antibiotics. Chem. Biodivers..

[62] Farhadi K., Rajabi E., Varpaei H.A., Iranzadasl M., Khodaparast S., Salehi M. (2024). Thymol and Carvacrol Against *Klebsiella*: Anti-Bacterial, Anti-Biofilm, and Synergistic Activities—A Systematic Review. Front. Pharmacol..

[63] Peter S., Sotondoshe N., Aderibigbe B.A. (2024). Carvacrol and Thymol Hybrids: Potential Anticancer and Antibacterial Therapeutics. Molecules.

[64] Marchese A., Orhan I.E., Daglia M., Barbieri R., Di Lorenzo A., Nabavi S.F., Gortzi O., Izadi M., Nabavi S.M. (2016). Antibacterial and Antifungal Activities of Thymol: A Brief Review of the Literature. Food Chem..

[65] Jo E.-R., Oh J., Cho S.I. (2022). Inhibitory Effect of Thymol on Tympanostomy Tube Biofilms of Methicillin-Resistant Staphylococcus aureus and Ciprofloxacin-Resistant *Pseudomonas aeruginosa*. Microorganisms.

[66] Ma Z., Qian C., Zhong Z., Yao Z., You C., Cao J., Zhou C., Ye J. (2025). Thymol Combined with SAEW for the Eradication of Mature *Pseudomonas aeruginosa* Biofilms and Reduction of Bacterial Virulence. Front. Microbiol..

[67] Niu C., Gilbert E.S. (2004). Colorimetric Method for Identifying Plant Essential Oil Components That Affect Biofilm Formation and Structure. Appl. Environ. Microbiol..

[68] Aquino S.F., Stuckey D.C. (2004). Soluble Microbial Products Formation in Anaerobic Chemostats in the Presence of Toxic Compounds. Water Res..

[69] Fang H.H.P., Xu L.-C., Chan K.-Y. (2002). Effects of Toxic Metals and Chemicals on Biofilm and Biocorrosion. Water Res..

[70] Burt S. (2004). Essential Oils: Their Antibacterial Properties and Potential Applications in Foods—A Review. Int. J. Food Microbiol..

[71] Liu T., Kang J., Liu L. (2021). Thymol as a Critical Component of *Thymus vulgaris* L. Essential Oil Combats *Pseudomonas aeruginosa* by Intercalating DNA and Inactivating Biofilm. LWT.

[72] Chatterjee B., Vittal R.R. (2021). Quorum Sensing Modulatory and Biofilm Inhibitory Activity of *Plectranthus barbatus* Essential Oil: A Novel Intervention Strategy. Arch. Microbiol..

[73] Merghni A., Haddaji N., Bouali N., Alabbosh K.F., Adnan M., Snoussi M., Noumi E. (2022). Comparative Study of Antibacterial, Antibiofilm, Antiswarming and Antiquorum Sensing Activities of *Origanum vulgare* Essential Oil and Terpinene-4-ol against Pathogenic Bacteria. Life.

[74] Vrenna G., Artini M., Ragno R., Relucenti M., Fiscarelli E.V., Tuccio Guarna Assanti V., Papa R., Selan L. (2021). Anti-Virulence Properties of Coridothymus capitatus Essential Oil against *Pseudomonas aeruginosa* Clinical Isolates from Cystic Fibrosis Patients. Microorganisms.

[75] Noumi E., Ahmad I., Adnan M., Patel H., Merghni A., Haddaji N., Bouali N., Alabbosh K.F., Kadri A., Caputo L. (2023). *Illicium verum* L. (Star Anise) Essential Oil: GC/MS Profile, Molecular Docking Study, In Silico ADME Profiling, Quorum Sensing, and Biofilm-Inhibiting Effect on Foodborne Bacteria. Molecules.

[76] Ghannay S., Aouadi K., Kadri A., Snoussi M. (2022). GC-MS Profiling, Vibriocidal, Antioxidant, Antibiofilm, and Anti-Quorum Sensing Properties of *Carum carvi* L. Essential Oil: In Vitro and In Silico Approaches. Plants.

[77] Fekry M., Yahya G., Osman A., Al-Rabia M.W., Mostafa I., Abbas H.A. (2022). GC-MS Analysis and Microbiological Evaluation of Caraway Essential Oil as a Virulence Attenuating Agent against *Pseudomonas aeruginosa*. Molecules.

[78] Khedhri S., Polito F., Caputo L., Manna F., Khammassi M., Hamrouni L., Amri I., Nazzaro F., De Feo V., Fratianni F. (2022). Chemical Composition, Phytotoxic and Antibiofilm Activity of Seven Eucalyptus Species from Tunisia. Molecules.

[79] European Pharmacopoeia Online. https://pheur.edqm.eu/subhome/11-8.

[80] Adams R.P. (2007). Identification of Essential Oil Components by Gas Chromatography/Mass Spectrometry.

[81] Andrews J.M. (2001). Determination of Minimum Inhibitory Concentrations. J. Antimicrob. Chemother..

[82] O’Toole G.A. (2011). Microtiter Dish Biofilm Formation Assay. JoVE.

[83] Savoia D., Zucca M. (2007). Clinical and Environmental *Burkholderia* Strains: Biofilm Production and Intracellular Survival. Curr. Microbiol..

[84] Filloux A., Ramos J.-L. (2014). Pseudomonas Methods and Protocols.

[85] Essar D.W., Eberly L., Hadero A., Crawford I.P. (1990). Identification and Characterization of Genes for a Second Anthranilate Synthase in *Pseudomonas aeruginosa*: Interchangeability of the Two Anthranilate Synthases and Evolutionary Implications. J. Bacteriol..

